# Quercetin ameliorates oxidative stress-induced apoptosis of granulosa cells in dairy cow follicular cysts by activating autophagy via the SIRT1/ROS/AMPK signaling pathway

**DOI:** 10.1186/s40104-024-01078-5

**Published:** 2024-09-05

**Authors:** Hongwei Duan, Fang Wang, Ke Wang, Shuai Yang, Rong Zhang, Chen Xue, Lihong Zhang, Xiaofei Ma, Xianghong Du, Jian Kang, Yong Zhang, Xingxu Zhao, Junjie Hu, Longfei Xiao

**Affiliations:** 1 College of Veterinary Medicine, Gansu Agricultural University, Lanzhou, 730070 Gansu China; 2 Gansu Key Laboratory of Animal Generational Physiology and Reproductive Regulation, Lanzhou, 730070 Gansu China; 3 Gansu Institute of Animal Husbandry and Veterinary, Pingliang, 744000 Gansu China; 4 School of Animal Science and Technology, Guangdong Polytechnic of Science and Trade, Guangzhou, 510640 Guangdong China; 5 Animal Science and Technology College, Beijing University of Agriculture, Beijing, 102206 China

**Keywords:** Apoptosis, Autophagy, Follicular cyst, Oxidative stress, Quercetin

## Abstract

**Background:**

Follicular cysts contribute significantly to reproductive loss in high-yield dairy cows. This results from the death of follicular granulosa cells (GCs) caused by oxidative stress. Quercetin is known to have significant antioxidant and anti-apoptotic effects. However, the effect of quercetin on follicular cysts has yet been elucidated. Therefore, this study aimed to explore the anti-oxidant and anti-apoptosis effects and potential molecular mechanisms of quercetin in H_2_O_2_-induced primary cow GCs and 3-nitropropionic acid (3-NPA)-induced mouse model of oxidative stress and thus treat ovarian cysts in dairy cows.

**Results:**

In this study, compared with estrus cows, cows with follicular cysts showed heightened levels of oxidative stress and increased follicular cell apoptosis, while autophagy levels were reduced. A model of oxidative stress was induced in vitro by H_2_O_2_ and showed significant increases in apoptosis together with reduced autophagy. These effects were significantly ameliorated by quercetin. Effects similar to those of quercetin were observed after treatment of cells with the reactive oxygen species (ROS) inhibitor *N*-acetylcysteine (NAC). Further investigations using chloroquine (autophagy inhibitor), rapamycin (autophagy activator), selisistat (SIRT1 inhibitor), and compound C (AMPK inhibitor) showed that chloroquine counteracted the effects of quercetin on oxidative stress-induced apoptosis, while rapamycin had the same effect as quercetin. In addition, the SIRT1/AMPK pathway inhibitors antagonized quercetin-mediated mitigation of the effects of oxidative stress on increased apoptosis and reduced autophagy. Consistent with the results in vitro, in mouse ovarian oxidative stress model induced by 3-NPA, quercetin activated autophagy through the SIRT1/AMPK signaling pathway, while alleviating oxidative stress damage and inhibiting apoptosis in mouse ovaries.

**Conclusions:**

These findings indicate that quercetin can inhibit apoptosis in GCs and restore ovarian function by activating autophagy through the SIRT1/ROS/AMPK signaling pathway, suggesting a new direction for the treatment of ovarian follicular cysts in high-yield dairy cows.

## Introduction

In dairy cows, follicular cysts are generally defined as follicular structures at least 2.5 cm in diameter that persist for a minimum of 10 d without developing into a corpus luteum [1, 2]. The incidence of ovarian cysts has been reported to be from 6% to 19%, with multiparous cows having a higher incidence (39%) of ovarian cysts than primiparous cows (11%) [2–4]. Follicular cysts constituted about 40% of all cysts of ovary [5]. Therefore, the development of follicular cysts is an important reason for breeding failure in dairy cows, leading to economic losses. At present, although the pathogenesis of follicular cysts is not fully understood, the apoptotic death of follicular cells is known to play a significant role in the process [6, 7]. It has been found that excessive accumulation of reactive oxygen species (ROS) during follicular development may be a key contributor to ovarian cell apoptosis [8, 9]. During normal follicular development, granulosa cells (GCs) secrete hormones and proteins to provide nutrients for oocyte development and ovulation, during which the redox balance of the follicular internal environment is tightly controlled [10–12]. The accumulation of ROS induces follicular cell apoptosis and adversely affects oocyte maturation, suggesting that disrupted redox homeostasis in the internal environment of these cells may be linked with the development of follicular cysts [13]. Therefore, targeting ROS accumulation and apoptosis in these cells may be effective directions for the development of drugs for treating follicular cysts.

Autophagy is a dynamic process by which eukaryotic cells protect themselves against pathogen invasion and the deleterious effects of damaged intracellular organelles and misfolded proteins [14, 15]. It has been found that normal levels of autophagy contribute to the survival of cells, while both insufficient and excessive autophagy induced by stress will lead to cell death [16–19]. In a D-galactose-induced model of premature ovarian insufficiency (POI), oxidative stress in ovarian cells was found to inhibit autophagy, ultimately leading to GCs apoptosis [20]. In vitro, a model of oxidative stress induced in bovine GCs by H_2_O_2_ showed that insufficient autophagy increased GC apoptosis [21]. In addition, it has also been reported that ROS accumulation induces excessive autophagy of follicular cells, resulting in apoptosis [22, 23]. Therefore, autophagy plays a key role in apoptosis induced by oxidative stress, but little is known of the autophagy process and mechanisms in the GCs of follicular cysts. Recent studies have found that sirtuin 1 (SIRT1) regulates various aspects of follicular development, including the modulation of autophagy and mitigation of oxidative stress [24–28]. Inhibition of SIRT1 reduces the ovarian reserve and participates in the development and progression of polycystic ovary syndrome (PCOS) and premature ovarian failure (POF) [29, 30]. Restoration of SIRT1-mediated autophagy was found to inhibit apoptosis in GCs resulting from oxidative stress and restored their ability to secrete estradiol [21, 31]. AMP-activated protein kinase (AMPK) plays an important role in cellular energy metabolism and is regulated by SIRT1 [32, 33]. Studies have shown that reductions in SIRT1 expression inhibit AMPK, and reduce both the antioxidant capacity of the cell and inhibit autophagy [34, 35]. Meanwhile, phosphorylation of AMPK directly promotes autophagy and is related to ROS accumulation [36]. However, the involvement of the SIRT1/ROS/AMPK axis in autophagy in ovarian cells during oxidative stress requires further investigation.

Quercetin is a flavonoid compound that has been shown to have a variety of biological properties, including anti-inflammatory, anti-apoptotic, and antioxidant activities [37, 38]. Quercetin can inhibit the early apoptosis of bovine oocytes and promote the proliferation of GCs [39, 40]. In both POI and POF, it can also restore ovarian function by ameliorating damage caused by oxidative stress and reducing apoptosis [41, 42]. Quercetin has also been found to regulate autophagy, for instance, activating autophagy to alleviate intervertebral disc degeneration [43]. It also protects rat GCs from oxidative damage by activating autophagy [44]. However, whether quercetin has therapeutic potential for follicular cysts in dairy cows remains unknown.

Therefore, in this study, the mechanism underlying the effects of quercetin on follicular cysts was explored by examining the levels of autophagy, apoptosis, and oxidative stress in follicular cysts from dairy cows and normal GCs, and treating in vivo and in vitro models of oxidative stress with quercetin. The findings may provide new direction into the use of quercetin in the treatment of follicular cysts in dairy cows.

## Materials and methods

### Animal ethics and sample collection

All animal procedures used in this study were approved by the Animal Ethics Committee of Gansu Agricultural University (NO. GSAU-Eth-VMC-2021-045). Female Holstein cows from Gansu province, China, underwent diagnostic examinations for follicular cysts at 2 d intervals for at least 10 d. A positive diagnosis represented the presence of cystic follicular structures measuring ≥ 2.5 cm in diameter with a follicle wall ≤ 3 mm thickness, which did not ovulate or form corpus lutea, as shown by rectal and ultrasonographic examinations [1, 2]. Follicles having walls of over 3 mm thickness were considered likely to be luteal cysts and were discarded [45]. Non-cystic follicles were defined as those between 1.2–2.0 cm diameter, together with the presence of obvious estrus behavior. These examinations led to the selection of 7 cows with follicular cysts and 7 with normal estrus. Blood samples were collected from the tail vein, and serum was separated by centrifugation at 3,000 r/min for 10 min for the determination of serum hormone and oxidative stress levels. Ovaries were collected from 4 cows with follicular cysts and 4 normal-estrus cows after sacrifice. For histological analysis, the ovaries were fixed with 4% paraformaldehyde, paraffin-embedded, stained with hematoxylin–eosin (H&E), and evaluated histologically. Follicle contents were collected from the cystic and non-cystic follicles by aspiration, and was centrifuged to separate the follicular fluid and GCs at 1,200 r/min for 5 min. The follicular fluid was stored at −80 °C for the measurement of oxidative stress levels, and the GCs were washed twice by centrifugation at 1,200 r/min for 5 min each, and stored at −80 °C before analysis by Western blotting.

### Animal grouping and oxidative stress modeling

Forty SPF female mice aged 6–8 weeks and weighted 28–32 g (obtained from the Experimental Animal Center of the Lanzhou Veterinary Research Institute) were housed in a controlled environment with a fixed temperature of 23 ± 1 °C, humidity of 45% ± 5% and 12 h light/dark cycle. In addition, the mice had free access to water plus food throughout the study. All the mice were allocated to four groups, namely, the control group (*n* = 10), 3-nitropropionic acid (3-NPA, F035948, purity ≥ 98%, Fluorochem, Manchester, UK) group (*n* = 10), 3NPA + quercetin (HY-18085, purity ≥ 98.06%, MedChemExpress, Monmouth Junction, NJ, USA) group (*n* = 10), and the quercetin group (*n* = 10). The 3-NPA group received intraperitoneal injections of 3-NPA (20 mg/kg/d) for 42 d [22, 23]. The quercetin group was given quercetin (200 mg/kg/d) for 42 d. The 3NPA + quercetin group received quercetin (200 mg/kg/d) after injections of 3-NPA for 42 d. The control group was given normal saline. On the final day of drug administration, d 43, the forty mice received general anesthesia induced by intraperitoneal injection of 150 mg/kg pentobarbital sodium, and they were subsequently sacrificed. The left ovaries were frozen at −80 °C following for further analysis of protein and oxidative stress levels, whereas the right ovary was fixed with paraformaldehyde and glutaraldehyde solution for histological analyses.

### Cell culture

GCs from cows were isolated according to previously described methods [46]. Briefly, forty-two fresh ovaries obtained from the slaughterhouse were placed in a thermos flask containing normal saline at 37 °C and were transported to the laboratory within 4 h. The contents of the follicles were extracted using a 1-mm syringe and were washed twice with phosphate-buffered saline (PBS), followed by the addition of hyaluronidase (1 g/L) to digest and separate the GCs and oocytes, after which the mixture was filtered using a 0.074-nm filter. After centrifugation at 1,000 r/min for 5 min and washing with PBS, the GCs were seeded in DMEM/F12 medium (Gibco, Waltham, MA, USA) containing penicillin (50 IU/mL), streptomycin (50 µg/mL) and 10% (v/v) fetal bovine serum (Gibco). The medium was replaced every 2 d. Cells grew to 80% confluency before treatment. Prior to treatment, the GCs were cultured in serum-free medium for 12 h.

### Cell treatment

The GCs were treated with H_2_O_2_ (50, 100 or 200 µmol/L) and/or quercetin (5, 10 or 20 µmol/L) for 3 h. The doses of H_2_O_2_ and quercetin were based on previous studies [21, 47]. For inhibition studies, the cells were treated with *N*-acetylcysteine (NAC, 5 mmol/L, MedChemExpress), chloroquine (CQ, 50 µmol/L, MedChemExpress), rapamycin (RAPA, 100 nmol/L, MedChemExpress), compound C (CC, 10 µmol/L, MedChemExpress), and selisistat (EX527, 10 µmol/L, MedChemExpress) for 2 h before treatment.

### Western blotting

Harvested samples were washed in PBS and processed using radioimmunoprecipitation assay (RIPA) buffer (Solarbio, Beijing, China) containing 1 mmol/L phenylmethylsulfonyl fluoride (Solarbio). Protein concentrations were determined using the BCA Protein Assay Kit (Solarbio). Samples containing the same amount of protein were separated on SDS-PAGE and transferred to polyvinylidene fluoride (PVDF, 0.45-μm) membranes (Millipore, MA, USA). The membranes were blocked in TBST solution (Tris-buffered saline containing 0.1% Tween-20) with 5% bovine serum albumin (BSA) for 2 h at 25 °C. And then, they were incubated overnight at 4 °C with rabbit polyclonal antibody, namely, anti-Bcl-2 (1:1,000, 12789-1-AP, Proteintech, Wuhan, China), anti-Bax (1:1,000, 50599-2-Ig, Proteintech), anti-cleaved caspase3 (1:1,000, 19677-1-AP, Proteintech), anti-LC3B (1:2,000, ab192890, Abcam, Cambridge, UK), anti-BECN1 (1:500, bs-1353R, Bioss, Beijing, China), P-AMPK alphaThr172 (1:1,000, 2535, Cell Signaling Technology, Danvers, MA, USA), anti-AMPK alpha (1:1,000, 5831 s, Cell Signaling Technology), anti-SIRT1 (1:1,000, PQA2569S, Abmart, Shanghai, China), and anti-β-actin (1:3,000, bs-0061R, Bioss). Next, the membranes were washed and incubated with horseradish peroxidase-conjugated goat anti-rabbit IgG (1:5,000, SA00001-2, Proteintech) for 1 h at 37 °C. The protein bands were visualized by addition of the ECL Plus Western blot detection reagent (Vazyme, Nanjing, China). Western blotting images were obtained using the Amersham Imager 600 chemiluminometer (GE Healthcare Bio-Sciences AB, Sweden). Densitometry of bonds was performed using Image J software.

### Hormone analysis

The serum concentrations of follicle-stimulating hormone (FSH), luteinizing hormone (LH), progesterone (P4), 17β-estradiol (E_2_), and insulin-like growth factor (IGF) were measured using enzyme-linked immunosorbent assay (ELISA) kits for cow FSH (IU/L), LH (ng/mL), E_2_ (pmol/L), P4 (ng/mL), and IGF (ng/mL) (Shfksc, Shanghai, China) according to the manufacturer’s instructions. The absorbances at 450 nm were measured in duplicate for each sample, and the absorbance values from the negative control wells (containing reaction solutions but no samples) were subtracted. The sensitivity of the ELISA kit for detecting FSH, LH, E_2_, P4 and IGF is < 0.1 IU/L, 1 ng/mL, 1.0 pmol/L, 0.1 ng/mL and 0.1 ng/mL, respectively. The coefficient of intraplate variation was less than 10%, and the coefficient of that was less than 15%.

### Hematoxylin–Eosin staining

Ovaries of dairy cows and mice were fixed for 24 h with 4% paraformaldehyde. The fixed samples were rinsed under flowing water, dehydrated using graded alcohol, transparentized through xylene, and embedded in paraffin, followed by the preparation of 4 μm-thick paraffin sections for Hematoxylin–Eosin staining. Olympus-DP73 optical microscope (Olympus, Tokyo, Japan) was used to evaluate the ovarian images.

### Evaluation of oxidative stress

The serum and follicular fluid of dairy cows and ovaries in mice were assessed for oxidative stress by analyzed the concentrations of ROS, the lipid peroxidation marker malondialdehyde (MDA), and total antioxidant capacity (T-AOC) following the instructions of marketed kit for ROS (BB-470515-100 T, Bestbio, Shanghai, China) together with MDA (E2009 and E2019, Applygen, Beijing, China) and T-AOC (E2006 and E2016, Applygen) kits.

### Immunofluorescence staining

Immunofluorescence staining was used to evaluate and localize FSHR and CYP19A1 in dairy cow GCs, and the expression of Cl-Cas3 and LC3II in the mouse ovaries [48, 49]. The cells and ovaries were fixed in 4% (v/v) paraformaldehyde, washed with cold PBS, treated with 0.1% (v/v) Triton X-100 for 30 min, and then incubated with 5% (w/v) BSA for 30 min. The samples were then incubated with polyclonal rabbit anti-FSHR (ab113421, 1:300, Abcam), anti-aromatase (ab18995, 1:300, Abcam), anti-LC3B (ab192890, 1:300, Abcam) and monoclonal mouse anti-Cl-Cas3 (bsm-33199 M, 1:300, Bioss) at 4 °C overnight. This was followed by incubation with CoraLite488-conjugated goat anti-rabbit IgG (H + L) secondary antibody (SA00013-2, 1:500, Proteintech) or CoraLite594-conjugated goat anti-mouse IgG (H + L) secondary antibody (SA00013-3, 1:500, Proteintech), and the nuclei were counterstained with 1 µg/mL 4′6-diamidino-2-phenylindole (DAPI, Solarbio). The negative control was incubated with PBS. Digital images were acquired using a fluorescence microscope (Apexbio, Beijing, China).

### Cell viability

The GC viability was detected using Cell Counting Kit-8 (CCK-8, IV08-100 T, Invigentech, Irvine, CA, USA) according to the manufacturer’s protocol. The primary GCs were seeded in 96-well plates at a density of 5 × 10^3^ cells/well. After treatment with different concentrations of H_2_O_2_ and quercetin for 3 h. Removing the supernatant, the cells were added 100 µL medium containing the CCK-8 assay reagent (10 µL) and incubated in the dark for 1 h at 37 °C. The optical density (OD) values were measured at a wavelength of 450 nm.

### Determination of total ROS in GCs

DCFH-DA was used to measure the levels of total ROS in GCs (S0033, Beyotime). To be specific, the primary GCs were seeded into 24-well plates at a density of 2 × 10^4^ cells/well and were treated with relevant instructions. Later, the cells were incubated (37 °C, 30 min) with 10 μmol/L DCFH-DA reagents in dark and rinsed in PBS gently. The cells were evaluated and imaged under fluorescence microscopy.

### Assessment of autophagosome flux

Autophagosome flux in the GCs was detected using the Ad-mCherry-GFP-LC3B assay (Beyotime). Briefly, the primary GCs were seeded in 6-well plates at a density of 1.5 × 10^5^ cells/well. The GCs were transfected with Ad-mCherry-GFP-LC3B for 24 h according to the protocol. The treatments were completed, GCs were foxed in paraformaldehyde solution, subjected to DAPI staining to detect the nucleus. And then, the mCherry and GFP fluorescent spots were analyzed. When autophagy occurs, mCherry-GFP-LC3B aggregates on the autophagosome membrane, presenting as yellow dots.

### Transmission electron microscopy (TEM)

Ovary samples were fixed in glutaraldehyde solution (2.5%) and subjected to a third fixation (1 h, 4 °C) in 1% osmium tetroxide, prior to rehydration, embedding. Samples were cut into sections (50 μm), stained with aqueous uranyl acetate and lead citrate. The autophagy in the mouse GCs were assessed by transmission electron microscope (Hitachi H-7500, Hitachi Ltd., Tokyo, Japan).

### Data analysis

All data are presented as the mean ± SD. GraphPad Prism 9 software (GraphPad Software, San Diego, CA, USA) was used for data analysis. Student’s *t* test was used to compare the differences between two groups, and multiple comparisons were analyzed via one-way analysis of variance. Statistical significance was defined as *P* value < 0.05.

## Results

### Levels of serum hormones in follicular cysts of dairy cows

**Fig. 1:**
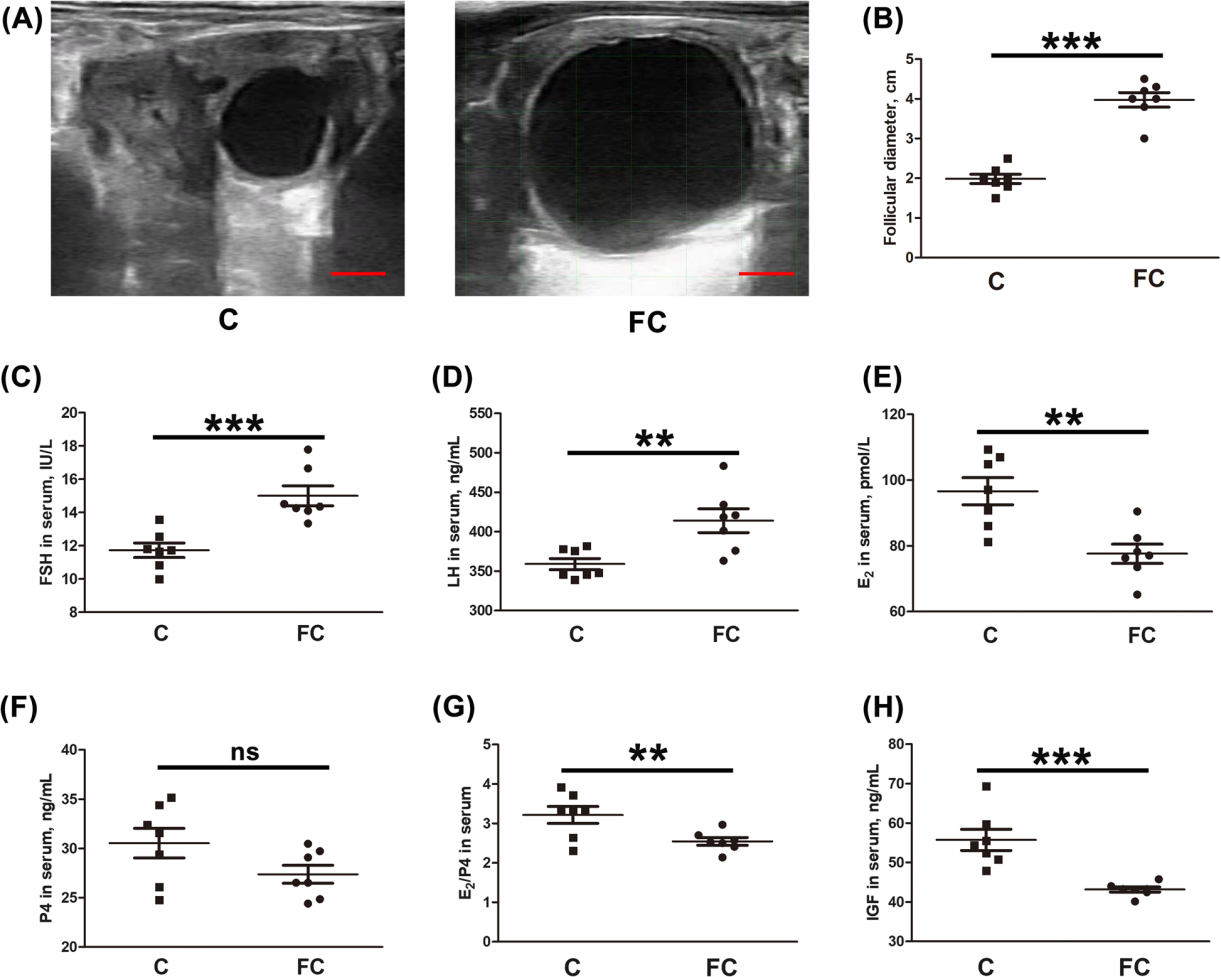
Levels of serum hormones in follicular cysts of dairy cows. **A** Ultrasonographic images of follicles from estrus cows and those with follicular cysts; scale bar = 1 cm. **B** The diameters of estrus and cystic follicles. **C** FSH, **D** LH, **E** E_2_, **F** P4, **G** E_2_/P4, and **H** IGF levels in the serum of estrus and follicular-cyst dairy cows (*n* = 7). C, estrus dairy cows; FC, follicular cyst dairy cows. Data are shown as mean ± SD; compared with the estrus group, ^**^P < 0.01, ^***^P < 0.001, ns, not significant

First, changes in the follicle diameter were evaluated in estrus cows and those with follicular cysts. Compared with the estrus cows, it was observed that the follicle diameter increased significantly when cows had follicular cysts. The mean diameter of the follicles in the estrus cows was 1.99 ± 0.12 cm, while the mean diameter in cows with follicular cysts was 3.97 ± 0.18 cm (Fig. 1A and B). The serum hormone levels in cows with follicular cysts were also significantly changed, seen by increased FSH (Fig. 1C, 11.73 ± 0.44 IU/L in estrus and 15.00 ± 0.44 IU/L in follicular cyst cows) and LH (Fig. 1D, 359.02 ± 6.99 ng/mL in estrus and 413.97 ± 15.03 ng/mL in follicular cyst cows), together with significantly decreased E_2_ (Fig. 1E, 96.64 ± 4.14 pmol/L in estrus and 77.60 ± 2.93 pmol/L in follicular cyst cows), E_2_/P4 (Fig. 1G, 3.22 ± 0.21 in estrus and 2.55 ± 0.10 in follicular cysts) and IGF (Fig. 1H, 55.75 ± 2.67 ng/mL in estrus and 43.17 ± 0.64 ng/mL in follicular cyst cows). There was no change in the P4 levels, shown in Fig. 1F (27.38 ± 0.91 ng/mL in estrus and 30.54 ± 1.51 ng/mL in follicular-cyst cows).

### Both apoptosis and oxidative stress increase in GCs from dairy cows with follicular cysts

**Fig. 2:**
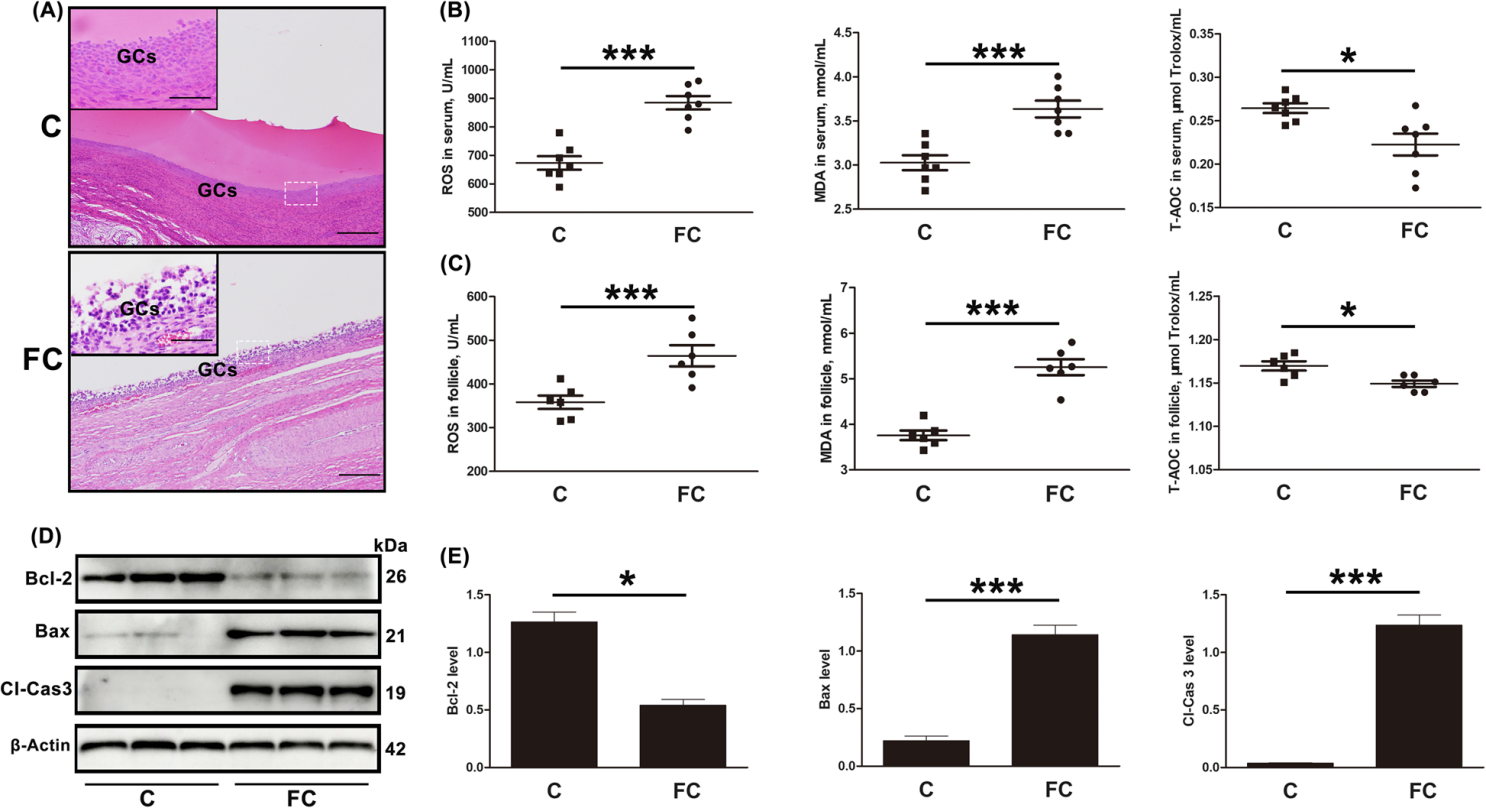
Increased levels of apoptosis and oxidative stress in GCs from dairy cows with follicular cysts. **A** HE staining showing histological changes in estrus and cystic follicles; Original magnification = 200 and 400 × ; scale bars = 100 and 50 μm. **B** The ROS, MDA, and T-AOC levels in the sera of estrus and follicular-cyst dairy cows. **C** The ROS, MDA, and T-AOC levels in the fluid from estrus and cystic follicles. **D** and **E** Relative expression of apoptosis-related proteins (Bcl-2, Bax, and Cl-Cas3) in GCs of estrus and cystic follicles, shown by Western blotting (*n* = 3). C, estrus dairy cows; FC, follicular-cyst dairy cows. The loading control was β-actin, and data are presented as mean ± SD. ^*^P < 0.05, ^***^P < 0.001

As illustrated in Fig. 2A, the GCs distribution in normal follicles was neat and compact, while that in cystic follicles was chaotic and scattered. Evidence indicates that apoptosis induced by oxidative stress plays an important role in the development of follicular cysts. Compared with estrus cows, the levels of ROS and MDA in the serum and follicular fluid of cows with cysts were significantly increased, while the levels of T-AOC were significantly decreased (Fig. 2B and C). In addition, as shown in the Fig. 2D and E, the level of Bcl-2 was significantly decreased in cystic follicular GCs, while those of Bax and CL-Cas3 were increased significantly. These findings suggest a relationship between oxidative stress and apoptosis in GCs from follicular cysts.

### Quercetin inhibits apoptosis in GCs induced by oxidative stress

**Fig. 3:**
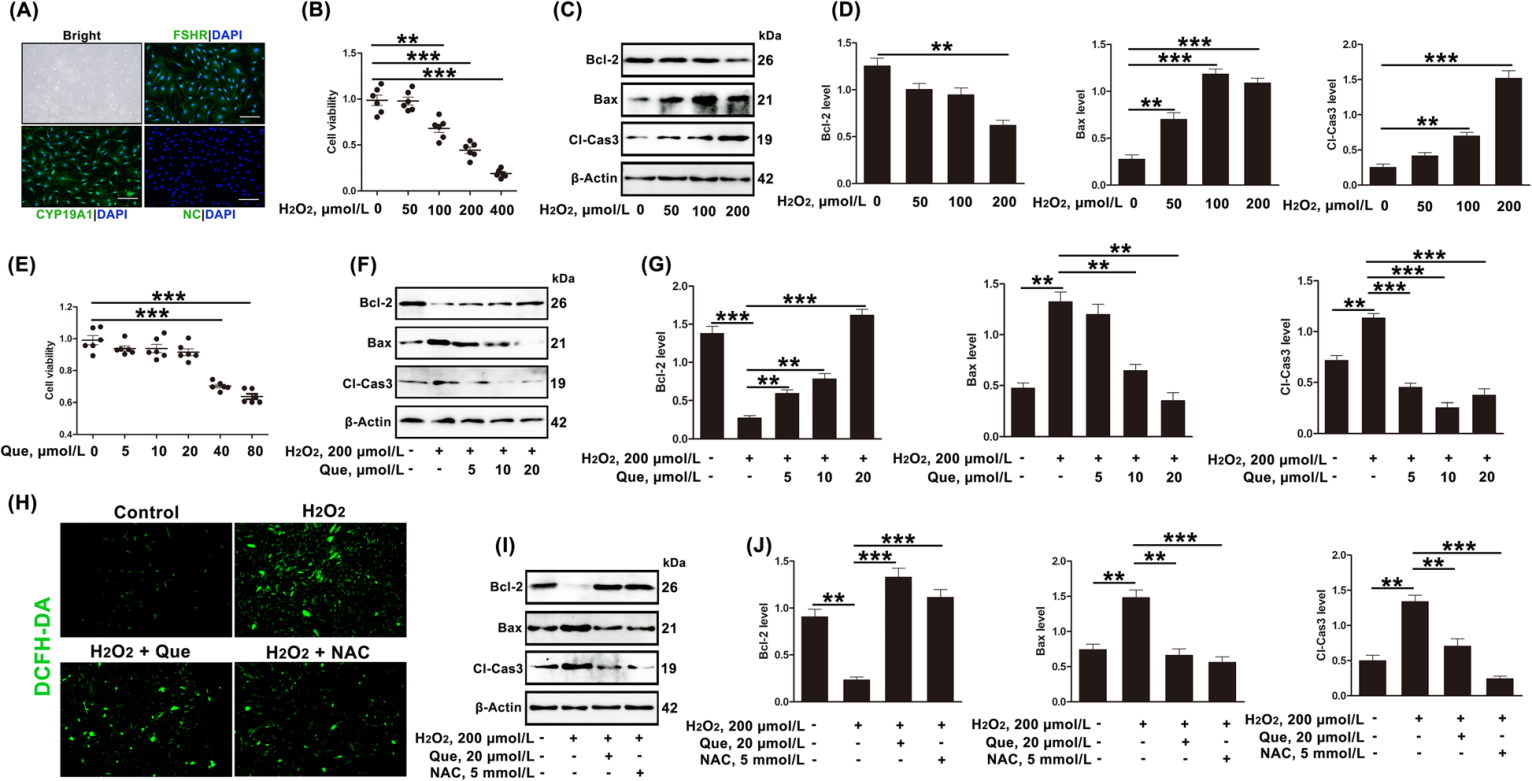
Quercetin inhibits oxidative stress-induced apoptosis in GC through antioxidant effects. **A** Immunofluorescence staining of FSHR and CYP19A1 were used to identify GCs in vitro, and PBS was used to replace the antibody in the NC group; scale bar = 100 μm. NC, negative control. **B** GC viability after treatment with H_2_O_2_ (50, 100, 200, 400 µmol/L) for 3 h (*n* = 6). **C** and **D** Relative protein expression of apoptosis-associated proteins (Bcl-2, Bax, and Cl-Cas3) in GCs treated with H_2_O_2_ (50, 100, 200 µmol/L), shown by Western blotting. **E** GC viability after treatment with quercetin (5, 10, 20, 40, 80 µmol/L) for 3 h (*n* = 6). **F** and **G** Relative protein expression of Bcl-2, Bax, and Cl-Cas3 in GCs treated with H_2_O_2_ (200 µmol/L) and/or quercetin (5, 10 and 20 µmol/L), shown by Western blotting. **H** Total ROS levels in GCs treated with H_2_O_2_ (200 µmol/L) and/or quercetin (20 µmol/L)/NAC (5 mmol/L), measured by DCFH-DA; scale bar = 100 μm. **I** and **J** Relative protein expression of Bcl-2, Bax, and Cl-Cas3 in GCs treated with H_2_O_2_ (200 µmol/L) and/or quercetin (20 µmol/L)/NAC (5 mmol/L), shown by Western blotting. Que, quercetin; NAC, *N*-acetylcysteine. The loading control was β-actin, and data are presented as mean ± SD. ^*^P < 0.05, ^**^P < 0.01, ^***^P < 0.001

In vitro modeling is a useful means of demonstrating the association between oxidative stress and apoptosis. Firstly, the expression of FSHR and CYP19A1 indicated that the cultured cells were GCs (Fig. 3A). The induction of oxidative stress in cells by H_2_O_2_ treatment is a well-established method [21, 44, 50]. As shown in Fig. 3B–D, after exposure of GCs to H_2_O_2_ (200 µmol/L), both cell viability and the expression of Bcl-2 decreased, while the expression of Bax and Cl-Cas3 increased. These results indicate that oxidative stress is the important inducement of apoptosis of GCs in dairy cows.

Quercetin has a powerful antioxidant effect [41, 42, 44]. After adding quercetin (40 and 80 µmol/L), the viability of the GCs in vitro decreased significantly (Fig. 3E). Thus, the 5, 10 and 20 µmol/L doses of quercetin were selected for further experiments. In addition, quercetin restored the apoptosis induced by H_2_O_2_ (Fig. 3F and G). To assess whether the effects of quercetin on oxidative stress-mediated apoptosis were mediated by decreasing ROS accumulation, NAC (ROS inhibitor) was used. As expected, both quercetin and NAC decreased ROS accumulation and apoptosis in GCs (Fig. 3H–J). This evidence showed that quercetin inhibited apoptosis induced by oxidative stress in GCs through antioxidant effects.

### Quercetin inhibits apoptosis in dairy cow GCs induced by oxidative stress by activating autophagy

**Fig. 4:**
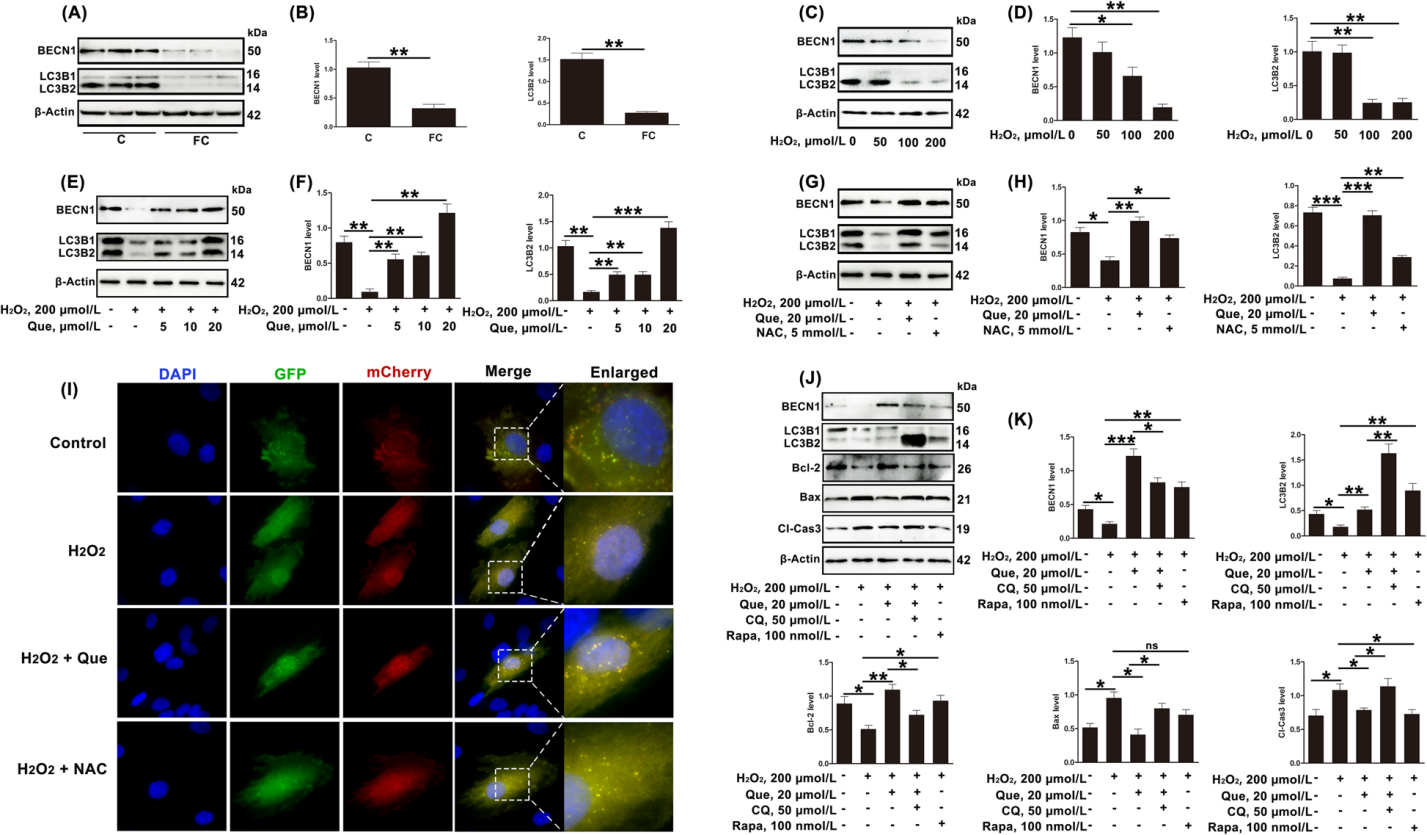
Quercetin inhibits oxidative stress-induced apoptosis in dairy cow GCs through autophagy activation. **A** and **B** Relative protein expression of autophagy-associated proteins (BECN1 and LC3B2) in GCs of estrus and cystic follicles, shown by Western blotting (*n* = 3). C, estrus dairy cows; FC, follicular-cyst dairy cows. **C** and **D** Relative protein expression of BECN1 and LC3B2 in GCs treated with H_2_O_2_ (50, 100, 200 µmol/L), shown by Western blotting. **E** and **F** Relative protein expression of BECN1 and LC3B2 in GCs treated with H_2_O_2_ (200 µmol/L) and/or quercetin (20 µmol/L), shown by Western blotting. **G** and **H** Relative protein expression of BECN1 and LC3B2 in GCs treated with H_2_O_2_ (200 µmol/L) and/or quercetin (20 µmol/L)/NAC (5 mmol/L), shown by Western blotting. **I** GCs treated with H_2_O_2_ (200 µmol/L) and/or quercetin (20 µmol/L)/NAC (5 mmol/L). After the transfection of GCs with adenovirus plus autolysosome quantitation via mCherry-GFP-LC3, autophagy levels were visually observed; scale bar = 50 μm. **J** and **K** Relative protein expression of BECN1 and LC3B2 were determined in GCs treated with H_2_O_2_ (200 µmol/L) and quercetin (20 µmol/L)/RAPA (100 nmol/L) and CQ (50 µmol/L) by Western blotting. Que, quercetin; NAC, *N*-acetylcysteine; RAPA, rapamycin; CQ, chloroquine. The loading control was β-actin, and data are presented as mean ± SD. ^*^P < 0.05, ^**^P < 0.01, ^***^P < 0.001, ns, not significant

It is reported that increasing autophagy flux under conditions of oxidative stress enables cell survival [21, 50]. In this study, compared with the estrus cows, the levels of the autophagy-related proteins LC3B2 and BECN1 were found to be significantly decreased in GCs from follicular cysts (Fig. 4A and B). In vitro, treatment of cells with H_2_O_2_ (100 and 200 µmol/L) decreased autophagy, suggesting that oxidative stress was an important cause of reduced autophagy in GCs (Fig. 4C and D). In addition, quercetin (5, 10, and 20 µmol/L) could restore the levels of autophagy (Fig. 4E and F), similar to the effects observed with the ROS inhibitor NAC (Fig. 4G–I). These results indicated that quercetin restored autophagy levels in GCs subjected to H_2_O_2_-induced oxidative stress through antioxidant effects.

The role of autophagy in the protective action of quercetin against oxidative stress-induced apoptosis was then investigated. The results showed that the effects of quercetin on related proteins of apoptosis and autophagy were significantly reversed after the addition of CQ (autophagy inhibitor). In contrast, similar to quercetin, treatment with RAPA (autophagy activator) mitigated changes in apoptosis and insufficient autophagy induced by the oxidative stress (Fig. 4J and K).

### Quercetin regulates autophagy and apoptosis by AMPK in dairy cow GCs

**Fig. 5:**
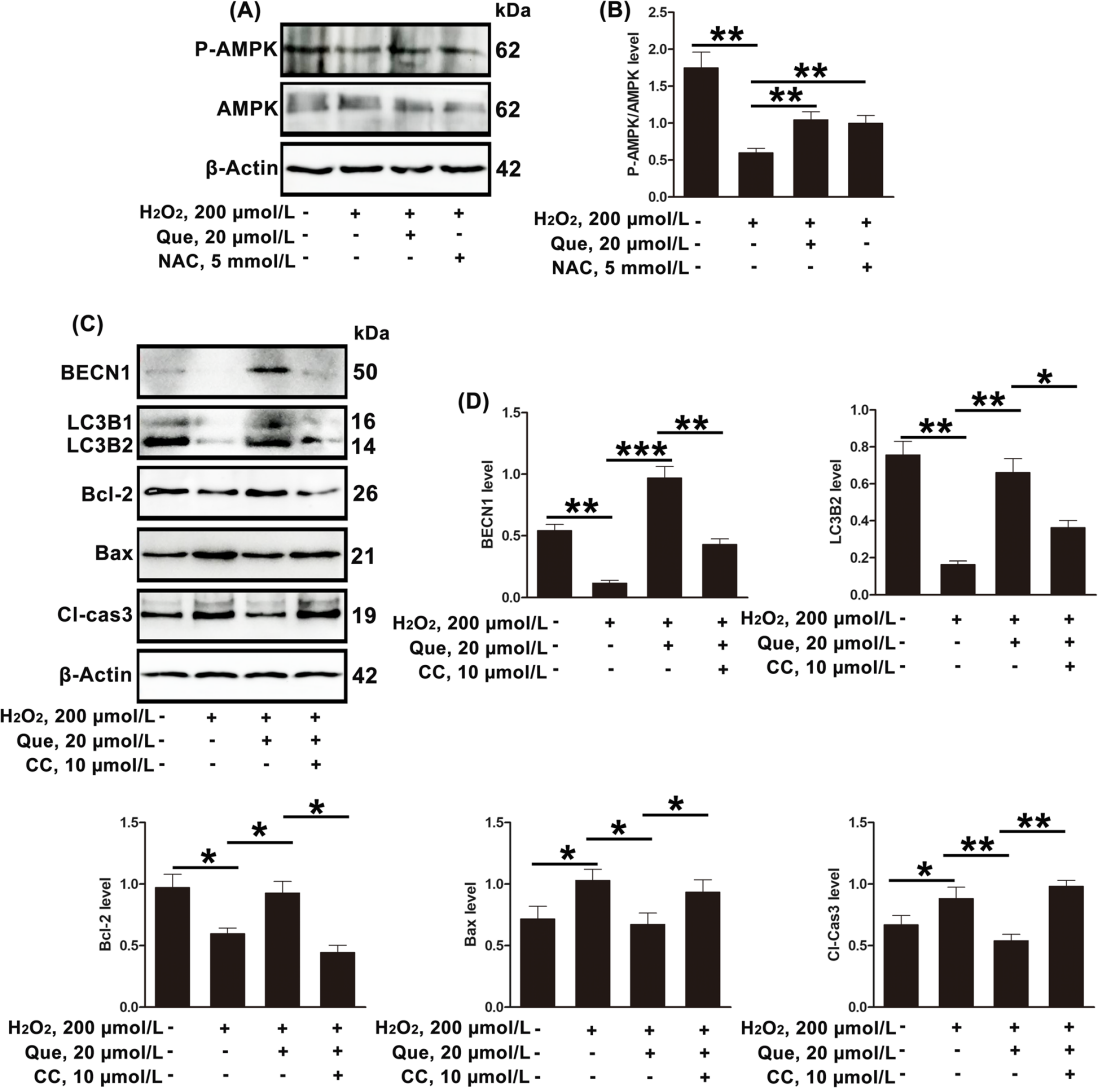
Quercetin inhibits GC apoptosis and activates autophagy by regulating the phosphorylation of AMPK. **A** and **B** Relative protein expression of P-AMPK and AMPK in GCs treated with H_2_O_2_ (200 µmol/L) and/or quercetin (20 µmol/L)/NAC (5 mmol/L), shown by Western blotting. **C** and **D** Relative protein expression of BECN1, LC3B2, Bcl-2, Bax, and Cl-Cas3 in GCs treated with H_2_O_2_ (200 µmol/L), quercetin (20 µmol/L), and/or CC (50 µmol/L), shown by Western blotting. Que, quercetin; NAC, *N*-acetylcysteine; CC, compound C. The loading control was β-actin, and data are presented as mean ± SD. ^*^P < 0.05, ^**^P < 0.01, ^***^P < 0.001

AMPK regulates many crucial cellular processes including autophagy [32]. Moreover, AMPK is involved in the regulation of ovarian function [51]. Therefore, in this study, the role of AMPK in the effects of quercetin on oxidative stress-induced apoptosis in ovarian cells was investigated. As shown in Fig. 5A and B, both quercetin and NAC mitigated the decrease in P-AMPK/AMPK induced by oxidative stress. Furthermore, to assess whether the AMPK plays a role in quercetin’s protection of GCs apoptosis and insufficient autophagy, CC (an AMPK inhibitor, 10 µmol/L) was added. The results showed that the regulatory effects of quercetin on apoptosis and autophagy were reversed by CC (Fig. 5C and D). To sum up, quercetin protected against apoptosis and insufficient autophagy by restoring the reductions in P-AMPK/AMPK caused by ROS accumulation and oxidative stress.

### Quercetin inhibits ROS accumulation and regulates AMPK levels to modulate autophagy and apoptosis in dairy cow GCs through SIRT1

**Fig. 6:**
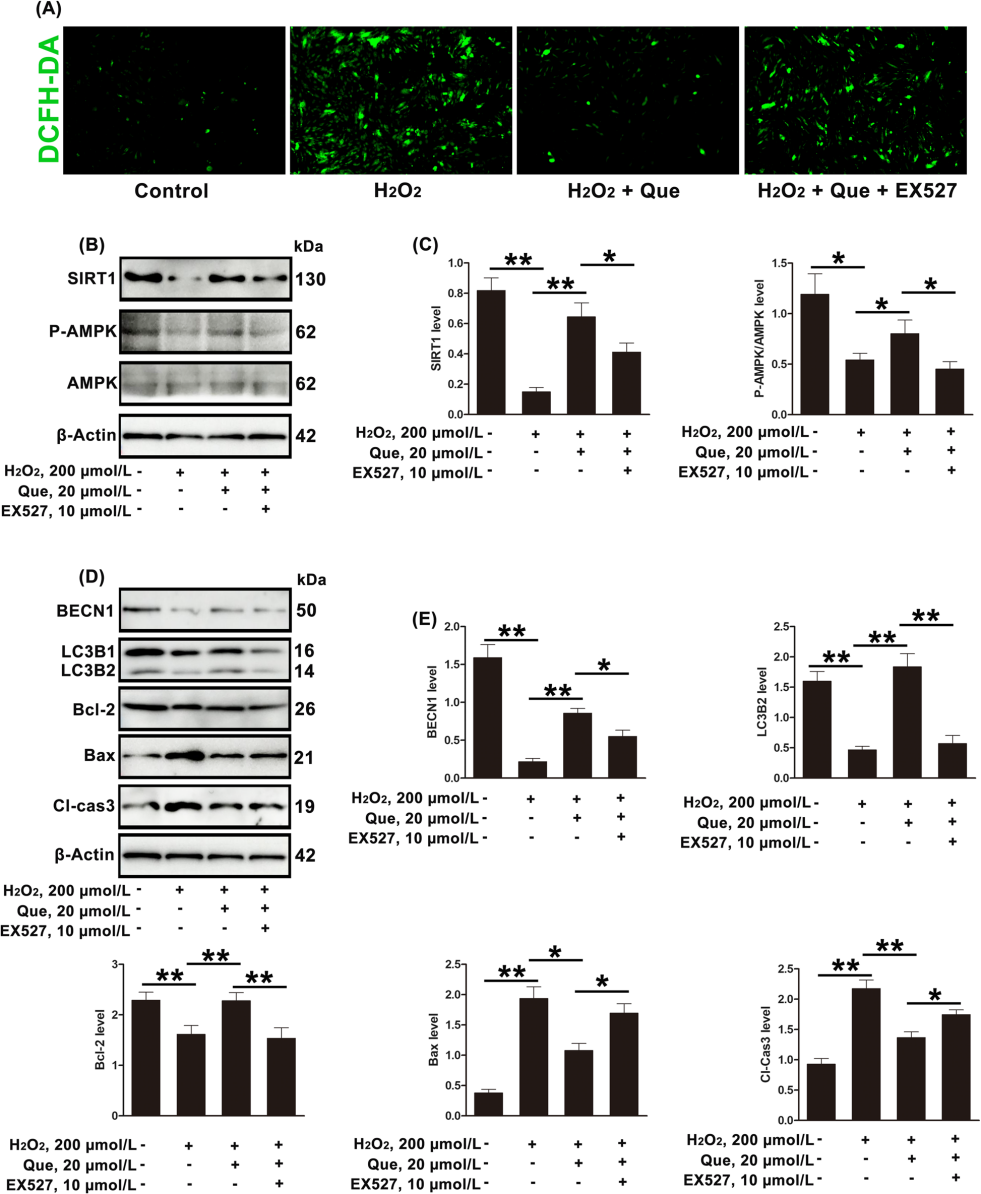
Quercetin inhibits ROS accumulation in GCs of dairy cows through SIRT1 and regulates autophagy through AMPK. **A** Total ROS levels in GCs treated with H_2_O_2_ (200 µmol/L), quercetin (20 µmol/L), and/or EX527 (10 µmol/L), measured by DCFH-DA; scale bar = 100 μm. **B** and **C** Relative protein expression of SIRT1, P-AMPK, and AMPK in GCs treated with H_2_O_2_ (200 µmol/L), quercetin (20 µmol/L), and/or EX527 (10 µmol/L), shown by Western blotting. **D** and **E** Relative protein expression of BECN1, LC3B2, Bcl-2, Bax, and Cl-Cas3 in GCs treated with H_2_O_2_ (200 µmol/L), quercetin (20 µmol/L), and/or EX527 (10 µmol/L), shown by Western blotting. Que, quercetin; EX527, selisistat. The loading control was β-actin, and data are presented as mean ± SD. ^*^P < 0.05, ^**^P < 0.01, ^***^P < 0.001

Recent studies have shown that SIRT1 promotes cell survival by regulating autophagy through AMPK [35]. In this study, the inhibition of ROS accumulation by quercetin was reversed by EX527 (SIRT1 inhibitor) (Fig. 6A). EX527 also reversed the regulatory effects of quercetin on P-AMPK/AMPK and autophagy, and the increase in apoptosis. These results indicated that quercetin inhibited ROS accumulation and restored P-AMPK/AMPK, autophagy and apoptosis by up-regulating the decline of SIRT1 induced by oxidative stress (Fig. 6B–E).

### Quercetin reduces oxidative stress-induced apoptosis in mouse GCs by activation of autophagy through the SIRT/ROS/AMPK pathway

**Fig. 7:**
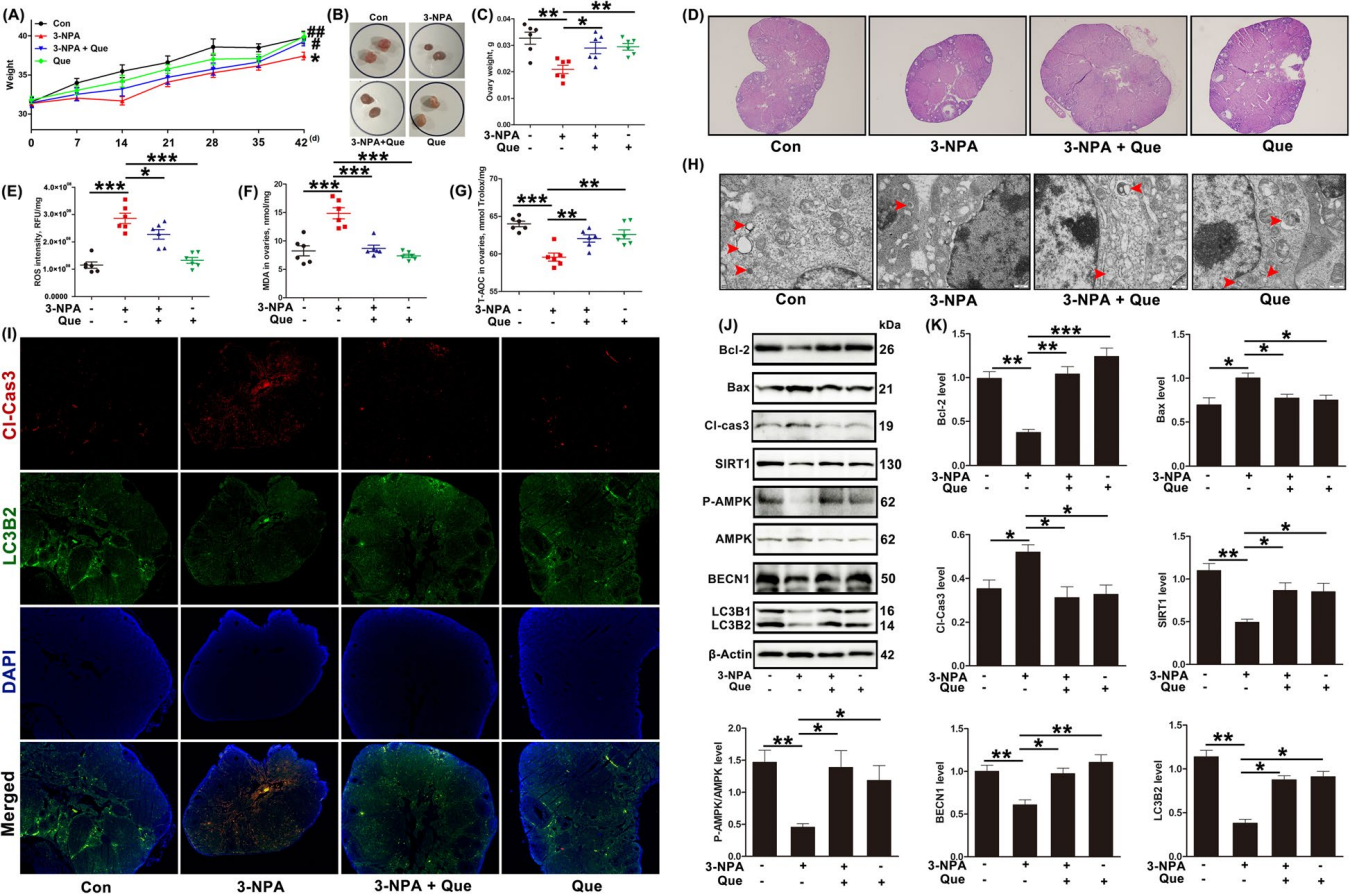
Quercetin reduces oxidative stress-induced apoptosis in mouse GCs by autophagy activation through the SIRT/ROS/AMPK signaling pathway. **A** Weights of 3-NPA- and/or quercetin-treated mice. **B** Ovaries of 3-NPA- and/or quercetin-treated mice. **C** Ovary weights of 3-NPA- and/or quercetin-treated mice. **D** Histological changes in the ovaries of 3-NPA- and quercetin-treated mice; original magnification = 40 × . **E** ROS, **F** MDA, and **G** T-AOC levels in the ovaries of 3-NPA- and quercetin-treated mice. **H** TEM images of autophagic structures in the GCs of 3-NPA- and quercetin-treated mice; scale bar = 500 nm. **I** Immunofluorescence staining of Cl-Cas3 and LC3B2 in the ovaries of 3-NPA- and quercetin-treated mice; original magnification = 40 ×. **J** and **K** Relative protein expression of Bcl-2, Bax, Cl-Cas3, SIRT1, P-AMPK, AMPK, BECN1, and LC3B2 in the ovaries of 3-NPA- and quercetin-treated mice, shown by Western blotting. The loading control was β-actin, and data are presented as mean ± SD. ^*^P < 0.05, ^**^P < 0.01, ^***^P < 0.001

Finally, the roles of quercetin on apoptosis in mouse ovaries after long-term injection of 3-NPA were evaluated. As shown in Fig. 7A–D, mice injected intraperitoneally with 3-NPA lost both body and ovarian weight, and recovered after being given quercetin. In addition, after injection of the mice with 3-NPA, the ovarian levels of ROS and MDA increased significantly, while those of T-AOC levels decreased significantly; all these levels recovered after quercetin treatment (Fig. 7E–G). Moreover, TEM showed that the number of double-membrane vesicular structures decreased in the 3-NPA group and increased significantly after quercetin treatment (Fig. 7H). Furthermore, immunofluorescence staining of Cl-Cas3 and LC3B2 showed that the expression of Cl-Cas3 increased while that of LC3B2 decreased in the oxidative stress model, with the expression of both proteins recovering after quercetin treatment (Fig. 7I). Lastly, Western blotting showed that quercetin could lower the levels of Bax and Cl-Cas3, while restoring the levels of Bcl-2, SIRT1, P-AMPK/AMPK, BECN1, LC3B2, in the mouse model of oxidative stress, consistent with the results obtained in the H_2_O_2_-treated GCs (Fig. 7J–K). To sum up, quercetin alleviated GC apoptosis induced by oxidative stress, which was associated with the activation of the SIRT1/ROS/AMPK axis regulating autophagy, thus contributing to the recovery of ovarian function.

## Discussion

Follicular cysts represent the main ovarian disorder leading to the loss of reproductive ability in high-yield dairy cows, which seriously restricts the development of dairy farming [4, 5, 52]. Studies have shown that redox imbalances in cows with follicular cysts damages ovarian function and eventually leads to infertility [53–55]. The results of the present study showed that, compared with cows with normal estrus, cows with follicular cysts showed increased levels of ROS and MDA in both the blood and follicular fluid, together with reduced T-AOC and increased apoptosis of GCs, suggesting that oxidative stress may be an important cause of apoptosis in GCs and eventual infertility in cows with follicular cysts. Quercetin is a natural flavonoid compound with a variety of biological functions, and can inhibit oxidative stress and maintain the function of the ovary [38, 40, 56]. In this study, both the in vivo and in vitro results showed that quercetin could protect GCs from oxidative stress, and that this effect was mediated by the activation of autophagy regulated by SIRT1/ROS/AMPK signaling pathway.

During follicular development in mammals, ROS produced by aerobic metabolism in follicular cells contributes to the formation of follicular cavities and ovulation [57]. An excessive accumulation of ROS results in a redox imbalance in the follicular cells, leading to their apoptosis and ultimately the loss of ovarian function [58, 59]. The in vitro and in vivo data in this study showed that excessive oxidative stress induced GC apoptosis, suggesting the importance of oxidative stress in the development of follicular cysts in dairy cows. Quercetin has been shown to have unique anti-oxidative properties, and has shown marked therapeutic effects in cyclophosphamide-induced POI models, letrozole-induced PCOS models, and in vitro rat GCs oxidative stress models [41, 42, 60]. In this study, quercetin was found to inhibit GCs apoptosis in dairy cows and the ovaries of mouse models of oxidative stress. In addition, similar to quercetin, the ROS inhibitor NAC could effectively reduce ROS accumulation in oxidative-stress models, while inhibiting apoptosis. These results suggest that the anti-apoptotic effect of quercetin may be mediated by inhibiting the accumulation of ROS and consequent oxidative stress.

Modulation of autophagy levels by oxidative stress is essential for cell survival [61, 62]. The present study found, for the first time, that autophagy levels were reduced in GCs from follicular cysts relative to those observed in cows with normal estrus. Autophagy levels were also found to be significantly reduced in both in vivo and in vitro models of oxidative stress, suggesting that oxidative stress-induced apoptosis of GCs may be related to insufficient autophagy. A recent study showed that quercetin can relieve senescent damage in rat GCs caused by oxidative stress through activating autophagy [44]. Furthermore, the present study found that the autophagy deficiencies induced by oxidative stress were restored by both quercetin and NAC, indicating that quercetin activates autophagy through its unique antioxidant effect. It has been reported that extracellular vesicles in bovine follicular fluid inhibit GC apoptosis by activating autophagy [63]. In addition, the levels of autophagy and apoptosis in GCs were significantly correlated during follicular development, and changes in autophagy determined the fate of GCs [64]. However, the role of autophagy in the reduction of oxidative stress-induced apoptosis by quercetin requires further investigation. It was found that, similar to quercetin, RAPA restored autophagy and alleviated oxidative stress-induced apoptosis, while the autophagy inhibitor CQ significantly inhibited the effects of quercetin. These results suggest that autophagy plays a key role in inhibiting the apoptosis of GCs induced by oxidative stress.

AMPK signaling plays a key role in autophagy regulation [32, 65]. Recent studies have found that the AMPK inhibitor CC can activate primordial follicles, as well as promoting follicular development and ovarian function [51, 66]. In addition, BEMC-exosomes restored autophagy by activating the P-AMPK/AMPK and alleviating damage to testicular tight junctions caused by D-galactose [67]. Both the in vitro and in vivo results of the present study showed that quercetin promoted P-AMPK/AMPK signaling by inhibiting ROS production, and the AMPK inhibitor CC reversed the regulatory effects of quercetin on oxidative stress-induced autophagy deficiency and increased apoptosis. Many studies have found that SIRT1 can activate AMPK to restore the cellular energy balance, regulate cell death and ovarian function [68–71]. In the ovary, SIRT1 is highly expressed in large follicles, and over-expression of SIRT1 can promote the development of follicles in mice [27, 72]. In addition, porcine follicular atresia is associated with reduced expression of SIRT1, indicating that the difference in the expression of SIRT1 in the ovary is closely related to ovarian function [73]. Increasing the levels of SIRT1 may represent an important way of treating ovarian diseases with drugs. In this study, quercetin promoted the expression of SIRT1 in the oxidative-stress model, and its inhibitory effects on oxidative stress-induced ROS accumulation, the ratio of P-AMPK/AMPK, and autophagy-regulated apoptosis were reversed by the SIRT1 inhibitor EX527, indicating a key role of SIRT1 in this process. These results confirmed that the deficiency of autophagy regulated by SIRT1/ROS/AMPK signaling pathway is key to the restoration of ovarian function by quercetin.

## Conclusions

In conclusion, the data demonstrate that quercetin can inhibit oxidative stress and apoptosis in the follicles of cows with follicular cysts, and that these effects are related to the level of autophagy regulated by the SIRT1/ROS/AMPK signaling pathway. The overall results of this study provide a new reference for the study of quercetin in infertility caused by ovarian disease in dairy cows, and reveal that the targeting of autophagy through the SIRT1/ROS/AMPK signaling pathway may be an effective treatment for oxidative stress-related ovarian diseases.

## Data Availability

All data generated or analyzed during this study are included in this published article.

## Abbreviations

3-NPA: 3-Nitropropionic acid
AMPK: AMP-activated protein kinase
BECN1: Beclin1
C: Estrus dairy cows
CC: Compound C
CCK-8: Cell Counting Kit-8
CQ: Chloroquine
DAPI: 4′6-Diamidino-2-phenylindole
DCFH: Dichlorodihydrofluorescein
E_2_: 17β-Estradiol
ELISA: Enzyme-linked immunosorbent assay
EX527: Selisistat
FC: Follicular cyst dairy cows
FSH: Follicle-stimulating hormone
GCs: Granulosa cells
H&E: Hematoxylin-eosin
IFG: Insulin-Like Growth Factor
LC3-II: LC3-phosphatidylethanolamine conjugate
LH: Luteinizing Hormone
MDA: Malondialdehyde
NAC: *N*-Acetylcysteine
P4: Progesterone
PBS: Phosphate-buffered saline
PCOS: Polycystic ovary syndrome
POF: Premature ovarian failure
POI: Premature ovarian insufficiency
PVDF: Polyvinylidene fluoride
RAPA: Rapamycin
RIPA: Radioimmunoprecipitation assay
ROS: Reactive oxygen species
SIRT1: Sirtuin 1
T-AOC: Total antioxidant capacity
TEM: Transmission electron microscopy
